# Isolation and characterization of phosphate-solubilizing bacterium *Pantoea rhizosphaerae* sp. nov. from *Acer truncatum* rhizosphere soil and its effect on *Acer truncatum* growth

**DOI:** 10.3389/fpls.2023.1218445

**Published:** 2023-07-14

**Authors:** Qinghua Ma, Shanwen He, Xing Wang, Zed Rengel, Lin Chen, Xinghong Wang, Shunxiang Pei, Xuebing Xin, Xiaoxia Zhang

**Affiliations:** ^1^Experimental Centre of Forestry in North China, National Permanent Scientifc Research Base for Warm Temperate Zone Forestry of Jiulong Mountain, Chinese Academy of Forestry, Beijing, China; ^2^State Key Laboratory of Tree Genetics and Breeding, Chinese Academy of Forestry, Beijing, China; ^3^Key Laboratory of National Forestry and Grassland Administration on Ecological Landscaping of Challenging Urban Sites, Shanghai Academy of Landscape Architecture Science and Planning, Shanghai, China; ^4^State Key Laboratory of Efficient Utilization of Arid and Semi-arid Arable Land in Northern China, Institute of Agricultural Resources and Regional Planning, Chinese Academy of Agricultural Sciences, Beijing, China; ^5^UWA School of Agriculture and Environment, The University of Western Australia, Perth, WA, Australia; ^6^Institute for Adriatic Crops and Karst Reclamation, Split, Croatia

**Keywords:** *Pantoea*, genome, phosphate-solubilizing bacteria, *Acer truncatum*, P accumulation

## Abstract

The *Acer truncatum* Bunge, widely distributed in North China, shows excellent tolerance to low-P soils. However, little information is available on potential phosphate-solubilizing bacterial (PSB) strains from the *A. truncatum* rhizosphere. The objectives of this work were to isolate and characterize PSB from *A. truncatum* rhizosphere soil and to evaluate the effect of inoculation with the selected strain on *A. truncatum* seedlings. The strains were characterized on the basis of phenotypic characteristics, carbon source utilization pattern, fatty acid methyl esters analysis, 16S rRNA gene and the whole-genome sequence. A Gram-negative and rod-shaped bacterium, designated MQR6^T^, showed a high capacity to solubilize phosphate and produce indole-3-acetic acid (IAA) and siderophores. The strain can solubilize tricalcium phosphate (TCP) and rock phosphate (RP), and the solubilization of TCP was about 60% more effective than RP. Phylogenetic analyses based on the 16S rRNA gene and whole-genome sequences revealed that strain MQR6^T^ formed a distinct phyletic lineage as a new species within the genus *Pantoea*. The digital DNA-DNA hybridization value between strain MQR6^T^ and the closely related strains was 19.5-23.3%. The major cellular fatty acids were summed feature 3 (C_16:1_ω7c and/or C_16:1_ω6c), summed feature 8 (C_18:1_ω6c and/or C_18:1_ω7c), C_14:0_, C_16:0_, and C_17:0_ cyclo. Several genes related to IAA production, phosphonate transport, phosphate solubilization and siderophore biogenesis were found in the MQR6^T^ genome. Furthermore, inoculation with the strain MQR6^T^ significantly improved plant height, trunk diameter, dry weight and P accumulation in roots and shoot of *A. truncatum* seedlings compared to non-inoculated control. These plant parameters were improved even further in the treatment with both inoculation and P fertilization. Our results suggested that MQR6^T^ represented a new species we named *Pantoea rhizosphaerae*, as a plant growth-promoting rhizobacterium that can solubilize inorganic P and improve growth of *A. truncatum* seedlings, emerging as a potential strategy to improve *A. truncatum* cultivation.

## 1 Introduction

Phosphorus (P) is an essential macronutrient for plant survival and reproduction, as a component of nucleic acids, membrane phospholipids and many energy-dependent metabolic processes (Vance et al., 2003). Phosphorus is frequently the most limiting element in soils because it rapidly forms insoluble complexes with cations and has low solubility and poor mobility in soils (Hinsinger, 2001; Rafique et al., 2022). The total amount of P is quite abundant in many soils, ranging from 0.02% to 0.5% (w/w), with an average of about 0.05% (w/w) (Son et al., 2006). However, plants absorb and assimilate P as inorganic (Pi) di- and monohydrogenphosphates; the average Pi concentration in the soil solution is 1 μM, which is below the Pi concentration needed for optimal plant growth (Hinsinger, 2001). Moreover, some assessments suggest world P reserves may last for only 50–200 years, which could result in a potential phosphate crisis (Herrera-Estrella and López-Arredondo, 2016). An ecologically friendly and economical approach to this problem may lie in the exploitation of the rhizosphere microbiome (De Zutter et al., 2022). Apart from P fertilization, soil P mobilization by microorganisms would be the only possible way to increase amounts of P available to plants (Etesami et al., 2021; Chouyia et al., 2022; Bouizgarne et al., 2023).

Rhizobacteria are plant-associated bacteria that colonize and persist in the proximity of roots or inside the root tissues (Backer et al., 2018). Phosphate-solubilizing bacteria (PSB) have the capacity to convert insoluble inorganic phosphates into soluble forms available to plants (Rafique et al., 2022). The principal mechanism for mineral P solubilization by PSB is associated with the production of low-molecular-weight organic acid anions, which through their hydroxyl and carboxyl groups chelate the phosphate-bound cations to liberate P in soluble forms (Hassan et al., 2019). Additionally, PSB are capable of producing physiologically active indole-3-acetic acid (IAA) and siderophores, which may have pronounced effects on plant growth (Luziatelli et al., 2020).

Strains from bacterial genera *Pantoea* have been reported as efficient PSB in the soil (Chen and Liu, 2019). The genus *Pantoea*, belonging to the family *Erwiniaceae* in the phylum *Proteobacteria* (Luziatelli et al., 2020), was first proposed by Gavini et al. (Gavini et al., 1989). The genus has been subsequently emended over the years as more species have been classified (Mergaert et al., 1993; Brady et al., 2010). The genus *Pantoea* showed a strong capacity of adaptation to a broad range of hosts and various environmental conditions, with strains isolated from plants, soil, fruits, seeds, the fruiting body of mushroom, humans, and animals (Castagno et al., 2011; Dutkiewicz et al., 2016; Luziatelli et al., 2020). Furthermore, many strains from *Pantoea* were efficient in solubilizing insoluble inorganic phosphate sources such as tricalcium phosphate (TCP) in the culturing medium (Chen and Liu, 2019; Li et al., 2020). Some isolates, including *P. ananatis* and *P. agglomerans*, were found to possess plant growth-promoting properties and reduce plant stress (Li et al., 2020; Luziatelli et al., 2020).

The genus *Acer* (family Aceraceae), commonly known as maple, comprises 129 species with many infraspecific taxa (Bi et al., 2016). These species are distributed in the temperate regions of Asia, Europe, northern Africa, and central and northern America (Bi et al., 2016; Wang et al., 2019). China (with 99 species reported) is considered to host the greatest diversity of the genus *Acer* (Bi et al., 2016). The *A. truncatum* Bunge is a forest tree species found in the north of China, showing excellent tolerance to P-deficiency stress (Wang et al., 2019). However, little information is available on potential PSB strains from the rhizosphere of *A. truncatum* growing on low-P soil. The purposes of this study were to isolate and characterize the PSB from rhizosphere soil of *A. truncatum* grown in the main production area in North China. In addition, the effects of inoculation of PSB with or without P fertilizer on root and shoot growth of *A. truncatum* seedlings and their P uptake were evaluated.

## 2 Materials and methods

### 2.1 Soil sampling and bacterial isolation

Rhizosphere soil samples of *A. truncatum* Bunge were taken from three sites in Jiulongshan Mountain Preserve, Beijing, People’s Republic of China (39°57′48″ N, 116°05′00″ E). At each sampling site, lateral roots of four *Acer* plants in the 10-30 cm soil layer were collected using a sterilized shovel and scissors. Samples of approximately 100 g of soil tightly adhering to lateral roots were collected in sterilized plastic bags, immediately placed on dry ice, and transferred to the laboratory for further work. Initial soil properties of the three sites were as follows: total N 1.3 g kg^−1^, total P 0.9 g kg^−1^, pH 8.1 (1:2.5, soil:water), Olsen-P 4.2 mg kg^−1^, available N (NH_4_^+^-N plus NO_3_^–^N) 17.3 mg kg^−1^, and exchangeable K 67 mg kg^−1^.

The serially diluted soil samples were plated on the TCP medium containing (per 1 liter): 5.0 g Ca_3_(PO_4_)_2_, 0.50 g (NH_4_)_2_SO_4_, 0.30 g NaCl, 0.30 g KCl, 0.30 g MgSO_4_, 0.03 g FeSO_4_, 0.03 g MgSO_4_, 0.50 g yeast extract, 10.0 g glucose, and 15.0 g agar. The PSB in the sampling rhizosphere were identified by clear halo zones around their colonies after 3 days of incubation at 30 °C. Experiments were performed in four replicates. The capacity of PSB to solubilize the water-insoluble phosphate was studied by the determination of solubilization index [the ratio of the total diameter (colony + halozone) and the colony diameter] (Li et al., 2020).

Single colonies with clear halos indicating P solubilization were selected and purified (Wang et al., 2020 and Bouizgarne et al., 2023). The purified strain designated MQR6^T^ was obtained and maintained (i) on tryptone soy agar (TSA) plates at 4°C in a refrigerator for further characterization and (ii) as suspensions supplemented with 30% (w/v) glycerol at ‐80°C. Strain MQR6^T^ was deposited at the China General Microbiological Culture Collection Center (CGMCC No. 23609^T^).

The reference strains *Pantoea vagans* DSM 23078^T^ and *Pantoea allii* DSM 25133^T^ were obtained from the Deutsche Sammlung von Mikroorganismen und Zellkulturen GmbH (DSMZ), and the strain *Pantoea ananatis* CICC 10283^T^ was obtained from the China Center of Industrial Culture Collection (CICC). These strains were cultured under the same conditions as described above for comparative analyses.

### 2.2 Morphological characterization

Colony morphology of purified bacterial isolate was studied by streaking on TSA medium, followed by incubation of the plates at 30°C for 24 h. Cell morphology was examined by light microscopy (model 50i, Nikon), and cellular morphology was observed by a scanning electron microscope (FEI Quanta 250 FEG, USA). Gram staining was performed using a bioMérieux Gram stain kit (Hangzhou Tianhe Microorganism Reagent Co.) according to the manufacturer’s instructions.

### 2.3 Phenotypic characterization

The temperature range for growth was determined in tryptone soy broth (TSB) liquid medium at 4, 10, 15, 20, 25, 28, 30, 37, 40, and 45°C. Growth at different pH values (3.0–12.0 at 1.0 pH unit increments) was evaluated in the TSB medium for 24 h, using the following buffer systems: pH 3.0–5.0, 0.1 M citric acid/0.1 M sodium citrate; pH 6.0–8.0, 0.1 M KH_2_PO_4_/0.1 M NaOH; and pH 9.0–12.0, 0.1 M NaHCO_3_/0.1 M Na_2_CO_3_. The range of NaCl concentrations for growth was determined in the TSB medium containing 0–10% NaCl at increments of 1% (w/v). Bacterial growth was measured by an increase in turbidity at 600 nm using a spectrophotometer.

Carbon source utilization tests, enzyme activity tests, and additional physiological and biochemical tests were performed using API-20NE, API 50CH (BioMérieux), and Biolog GEN III MicroPlate systems (Reis et al., 2004). The bacterial inoculation was performed according to the manufacturer’s instructions. The type strains of *P. vagans* DSM 23078^T^, *P. allii* DSM 25133^T^ and *P. ananatis* CICC 10283^T^ were used as reference strains. The results for API 20NE and API 50CH were obtained after 48 h of incubation as recommended by the manufacturer. When the Biolog system was used, strains were incubated on biological universal growth medium (Biolog) at 30 °C for 24 h. GEN III microplates were inoculated according to the manufacturer’s instructions and incubated at 30 °C for 22 h. Results were captured and analyzed based on an extensive species library in the Biolog GEN III database (Woźniak et al., 2019).

For cellular fatty acid analysis, cell mass of strain MQR6^T^ was harvested from TSA plates after incubation for 24 h at 30 °C. The fatty acid methyl esters were extracted and prepared according to the methods described by Sasser (1990). The fatty acids methyl ester mixtures were separated and analyzed on an Agilent GC-6890N gas chromatograph using the Sherlock Microbial Identification System with standard MIS Library Generation Software (version 6.0; Microbial ID Inc., Newark, DE, USA).

### 2.4 Phylogenetic 16S rRNA gene analysis

Genomic DNA was extracted using a Bacterial DNA Kit (Tiangen, Beijing, China) following the manufacturer’s instructions. The 16S rRNA gene was amplified by PCR using the universal primers 27F (5’-AGAGTTTGATCCTGGCTCAG-3’) and 1492R (5’-GGTTACCTTGTTACGACTT-3’) (He and Wan, 2022). and the purified PCR products were sequenced by Sangon Biotech (Shanghai, PR China). The 16S rRNA gene sequences were assembled by using the SeqMan package (DNAStar). The 16S rRNA gene sequence of strain MQR6^T^ was compared with the sequences available in the National Center for Biotechnology Information (NCBI) GenBank database (https://blast.ncbi.nlm.nih.gov/Blast.cgi) and EzBioCloud (www.ezbiocloud.net/identify) (Kim et al., 2012). Multiple alignments were carried out by using CLUSTAL_X software (Thompson et al., 1997). The phylogenetic trees were constructed using the neighbour joining (Saitou and Nei, 1987), maximum likelihood (Felsenstein, 1981) and maximum parsimony (Tamura et al., 2011) methods with the MEGA version 7.0 program (Kumar et al., 2016). The evolutionary distances were calculated using the Maximum Composite Likelihood Method (Tamura et al., 2004). Bootstrap values were calculated based on 1000 bootstrap replications in each case.

### 2.5 Complete genome sequencing and analysis

For genome sequencing of strain MQR6^T^, Illumina Hiseq TM2500 sequencing was performed at Shanghai Personal Biotechnology Co. (Beijing, PR China). The raw data were filtered and trimmed by AdapterRemoval (version 2.1.7) (Schubert et al., 2016). The trimmed reads were assembled using A5-miseq v20150522 with default parameters (Coil et al., 2015). CheckM v1.0.3 was used to estimate the completeness of the genome (Chen et al., 2020). Protein-coding open-reading frames were predicted by using Glimmer v3.02 (Delcher et al., 2007). Contigs longer than 1 kb and with read coverage of more than 10 were kept for further analysis. The G+C content of the chromosome was determined according to a draft genome sequence. The tRNA genes were predicted by tRNAscan-SE 94 (ver. 1.3.1) and the rRNA genes by Barrnap (0.9-dev) 95 (https://github.com/tseemann/barrnap) (Lowe and Eddy, 1997). Gene function prediction was performed by the Rapid Annotations using Subsystems Technology (RAST v.2.0) server (http://rast.nmpdr.org) (Overbeek et al., 2014) and eggNOG-mapper v2 (http://eggnog-mapper.embl.de) (Cantalapiedra et al., 2021). Metabolic pathways were analyzed by using the KEGG’s Blast KOALA service (Kanehisa et al., 2016).

The average nucleotide identity based on blase (ANIb) among the strain MQR6^T^ and related species was calculated using JspeciesWS online (Richter et al., 2016). The estimated digital DNA–DNA hybridization (dDDH) values among the strains were calculated by Genome-to-Genome Distance Calculator (GGDC2.0) with the alignment method of BLAST+ (Auch et al., 2010). The partial genome files were uploaded to the GGDC 2.0 web interface (http://ggdc.dsmz.de/ggdc.php#), and Formula 2 was used as recommended for the calculation of dDDH values. The proposed and generally accepted species boundary for ANIb and dDDH values are 95~96 and 70%, respectively (Meier-Kolthoff et al., 2013).

Two methods were used to construct phylogenetic trees of strain MQR6^T^ and the closely related *Pantoea* species. The first method used the classification workflows in Genome Taxonomy Database toolkit version 2.0.0 (GTDB-Tk) to identify and concatenate 120 single-copy bacterial marker genes (Chaumeil et al., 2020). The ML phylogenetic tree was established using IQ-TREE 2.2.0 program, SH-aLRT test, 1000 repeated ultrafast guidance, and ModelFinder to determine the best-fit model (Nguyen et al., 2015). The second method uploads the genome sequence data to the Type (Strain) Genome Server (https://tygs.dsmz.de/) (Meier-Kolthoff et al., 2022) for the whole-genome-based taxonomic analysis. The Bacterial Pan Genome Analysis (BPGA) pipeline was used for the pan-genome analyses of strain MQR6^T^ and the closely related *Pantoea* species (Chaudhari et al., 2016).

### 2.6 Quantification of P-solubilizing capacity

The ability of the strain to solubilize water-insoluble phosphate was measured in liquid media containing either TCP or powdered RP. The RP medium was modified from the TCP medium by adding 5 g L^-1^ of rock phosphate instead of TCP. One milliliter of MQR6^T^ culture (approximately 1×10^8^ cfu mL^-1^) was transferred to a 300-mL flask containing 100 mL of medium, followed by shaking (150 rpm) at 30 °C. The non-inoculated TCP and RP media were used as controls. Quadruplicate cultivations were conducted for each medium. The suspensions were sampled up to 96 hours at 12-hour intervals. At every sampling time, 3 mL of culture liquids was sampled and centrifuged at 5000 g (Anke LXJ-IIB) for 20 min to remove biomass and insoluble matter, and the supernatants were used for determination of pH and soluble P concentration. The pH value of the medium was measured with a pH meter. Phosphorus in the supernatant was determined by the molybdenum-blue method using a spectrophotometer at 700 nm (Watanabe and Olsen, 1965).

### 2.7 Quantification of IAA production

The secretion of plant growth hormone IAA by strain MQR6^T^ was measured by colorimetry. The test was performed in the presence and absence of L-tryptophan as the precursor of IAA. One milliliter of bacterial culture (approximately 1×10^8^ cfu·mL^-1^) was added to 100 mL TSB medium (with 5 mM L-tryptophan or without) in 250 mL Erlenmeyer flasks, and then incubated on a shaker (30 °C, 150 rpm) for 96 h. The suspensions were sampled at 12-hour intervals. The method to collect culture supernatant was the same as that in detecting P-solubilizing capacity. The production of IAA was screened by mixing 100 µL of bacterial suspension droplets with 100 µL of Salkowski reagent (50 mL 35% v/v HClO_4 +_ 1 mL 0.5 mol L^-1^ FeCl_3_) on a white ceramic plate based on the color change after 30 min of reaction in the dark at room temperature (Naqqash et al., 2016). Indole compounds react with Salkowski reagent to form a pink chromophore in absorbance at 530 nm using spectrophotometer (UV 3200, Shanghai, China). IAA concentrations were determined using a standard curve made from commercial IAA (Sigma), with the sterile medium as the blank.

### 2.8 Screening for siderophore production

Siderophore production was assayed qualitatively as described by Schwyn and Neilands (Schwyn and Neilands, 1987). Briefly, overnight culture of strain MQR6^T^ was spot-inoculated onto a chrome azurol S (CAS) agar plate and incubated for 5 days at 30° C. The basic principle underlying the test is that when a strong ligand (for example, siderophore) is added to a highly coloured dye-Fe^3+^ complex, the Fe^3+^-ligand complex is formed, and the release of free dye is accompanied by a colour change. When a strong chelator removes the iron from the dye, its color turns from blue to orange.

### 2.9 Plant inoculation experiments

The experiment was carried out in a greenhouse located at the Experimental Centre of Forestry in North China, Chinese Academy of Forestry, to evaluate the effects of strain MQR6^T^ on plant growth and nutrient uptake by *A. truncatum* seedlings. The soil was obtained from the *Acer* forest in Jiulongshan Mountain Preserve, with the properties as described above. The soil samples were air-dried, passed through a 2-mm sieve and filled into the pots at bulk density of 1.32 g cm^-3^. To ensure that the supply of other nutrients was adequate for plant growth, soil was supplemented with basal nutrients at the following rates (mg kg^−1^ soil): 200 N (NH_4_NO_3_), 50 Ca (as CaCl_2_), 150 K (as KCl), 28 Mg (as MgSO_4_), 4 Zn (as ZnSO_4_), and 1 Fe (as EDTA-Fe).

There were four treatments in the present study: (1) control, non-inoculated and without the application of P fertilizer (CK), (2) application of P fertilizer only, non-inoculated (P), (3) inoculation with MQR6^T^ only, without the application of P, and (4) inoculation with MQR6^T^ plus the application of P (MQR6+P). Monopotassium phosphate (KH_2_PO_4_) is a highly water-soluble inorganic salt, widely used as a P fertilizer in agricultural soils. The fertilizer KH_2_PO_4_ was used as P source, and the concentration was 50 mg P kg^-1^ soil.

The *Acer truncatum* seeds were surface-sterilized with sodium hypochlorite (1% w/v) for 30 min and then rinsed extensively with sterilized distilled water. The suspension of overnight bacterial culture (TSB) was diluted in sterile distilled water to a final concentration 10^8^ cfu mL^-1^, and the resulting suspensions were used to treat seeds and seedlings. The surface-sterilized seeds were dipped in the inoculum (containing 10^8^ cfu mL^-1^) for 15 min and then placed in pots containing 800 g of soil on 25 June 2021. Seeds dipped in medium not containing the strain were used for the non-inoculated treatments. A second inoculation was done at days 45 after seedling emergence at rates of 5 mL of bacterial suspension described above per pot. Plants were watered weekly to maintain 70–80% of field capacity.

Plants were harvested at 290 days after sowing on 10 April 2022. Plant height was recorded by measuring the length from soil surface to the tip of the main stem. Trunk diameter was measured at the base (5 cm from the ground) using a digital vernier caliper with an accuracy of 0.01 mm (Wuxi Kaibaoding Tools Co., Ltd., China). Chlorophyll content was read in the youngest fully-developed leaves using a chlorophyll meter (SPAD-502, Minolta, Osaka, Japan). The plants were separated into shoot and roots. The roots were kept in an icebox, transported to the lab, rinsed with water, and scanned by a scanner at resolution of 400 dpi. Root images were analyzed using WinRhizo Pro 2009b software (Regent Instruments Inc., Quebec, Canada) to calculate the root length and surface area. The shoots and roots were oven-dried at 105°C for 30 min and then at 70°C for 3 days to constant weight to determine dry weights and P uptake. P contents were assayed using the dry ashing digestion method.

### 2.10 Statistical analyses

One-way analysis of variance was performed using SAS statistical software (SAS 8.1, USA), and significant differences among means were assessed using Tukey’s test at 5% probability (*P ≤* 0.05).

## 3 Results

### 3.1 Morphological and physiological characteristics of MQR6^T^

**Figure 1:**
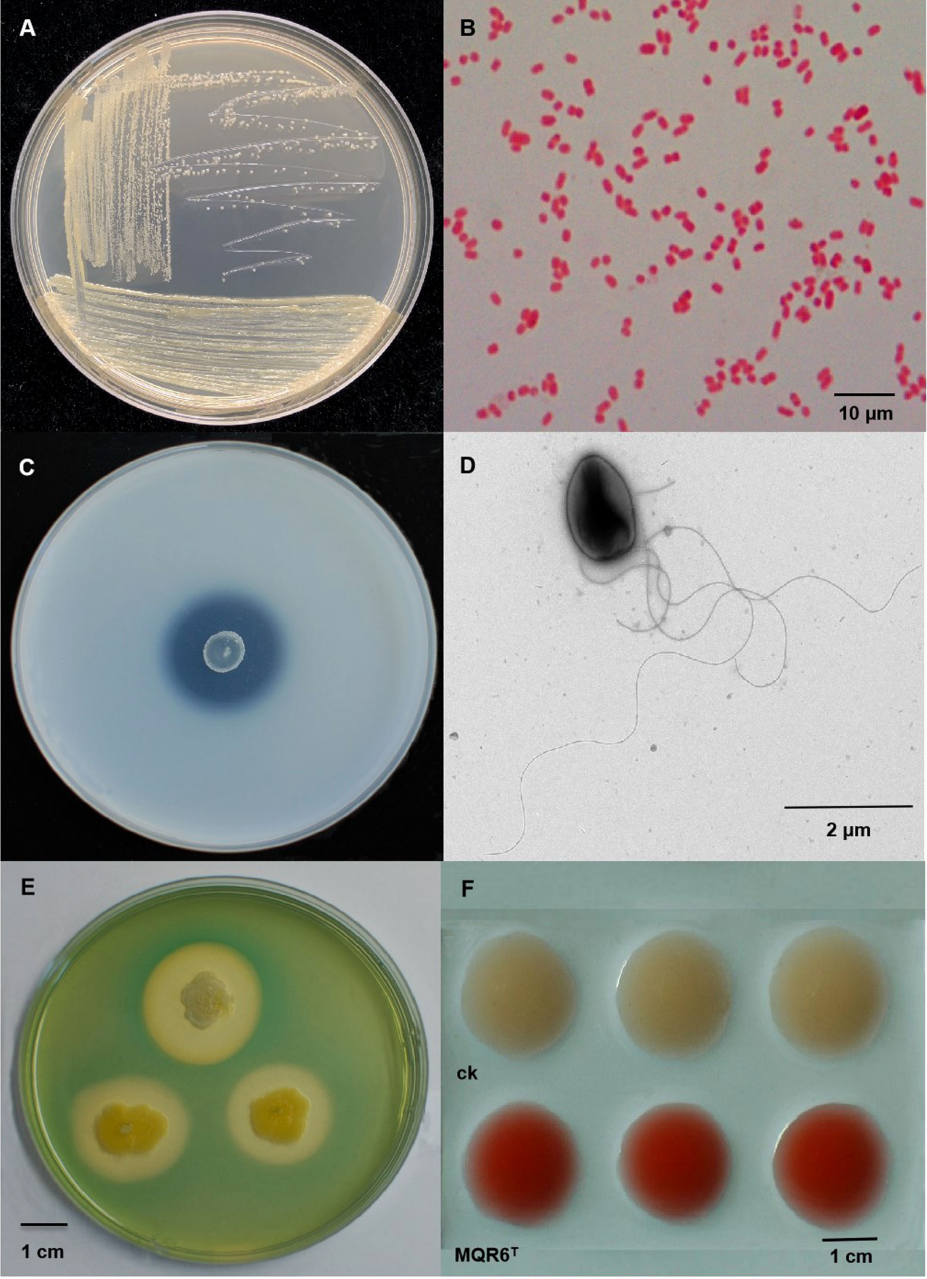
The morphological characteristics and plate assays of strain MQR6^T^. **(A)** The colony morphology on TSA plate; **(B)** The Gram-staining of the strain; **(C)** Halo zone on the agar medium containing TCP; **(D)** The cell morphology of the strain; **(E)** Halo zones of siderophore exudation on CAS plate; **(F)** Screening of IAA production with Salkowski reagent. TSA, tryptone soy agar; TCP, tricalcium phosphate; CAS, chromo azurol S; IAA, indole-3-acetic acid.

Colonies were circular, smooth, mucoid, convex with clear edges, and 0.8–2.0 mm in diameter after 24 h of incubation at 30°C on TSA plates (**Figure 1A**). Cells were rod-shaped, Gram-stain negative, single, non-spore-forming, measuring 0.5–1.4 μm×1.0–3.0 μm (**Figures 1B–D**). Bacterium features one or more long flagella (**Figure 1D**). Growth was found to occur at 10–40°C (optimum, 28–30°C) and at pH 4–11 (optimum, pH 7–9). In TSB medium, growth occurred in the presence of 0–7% (w/v) NaCl (optimum, 0–1%).

The phenotypic properties differentiating between strain MQR6^T^ and its closest phylogenetic neighbors are shown in **Table 1**. According to API 50CH tests, strain MQR6^T^ showed negative results with glycerin and sucrose, which were positive (or weakly positive) for *P. vagans* DSM 23078^T^, *P. allii* DSM 25133^T^ and *P. ananatis* CICC 10283^T^. Acid was produced from the fermentation of D-glucose, ribose, D-xylose, galactose, fructose, rhamnose, mannitol, N-acetylglucosamine, maltose, and trehalose by strain MQR6^T^ and the other three reference strains. With API 20NE, strain MQR6^T^ was negative for sodium citrate, L-arabinose, inositol, melibiose, and rhamnose but the other three reference strains were positive (at least weakly). Strain MQR6^T^ grew on D-maltose, but the other three reference strains did not.

**Table 1:** Differential phenotypic characteristics of strain MQR6^T^ and closely related strains in genus *Pantoea*.

Characteristic	MQR6^T^	*P. vagans* DSM 23078^T^	*P. allii* DSM 25133^T^	*P. ananatis* ATCC 19321^T^
Temperature range (°C)	10-40	10-42	10-42	10-42
Optimum temperature (°C)	28-30	30	30	30
pH range; Optimum	4-11; 7-9	4-11; 7-9	4-11; 7-9	4-11; 7-9
NaCl tolerance (% w/v)	0-7	0-7	0-7	0-7
Acid production				
Glycerin	−	W	+	+
Cellobiose	+	−	+	+
Adonitol	−	−	+	−
Lactose, raffinose, D-arabitol, sorbitol, *α*-methyl-D-mannosidase, melibiose	−	−	+	+
Sucrose	−	+	+	+
Sodium citrate, melibiose, L-arabinose	−	+	+	+
L-tryptophane	−	−	+	+
Inositol, rhamnose	−	+	W	W
Carbon resources (Biolog GEN III)				
D-melibiose, D-raffinose, dextrin, D-arabitol	−	−	+	+
D-maltose	+	−	−	−
Gentiobiose	−	−	+	−
*α*-D-lactose, sucrose	−	−	W	+
L-fucose	+	−	W	−
D-sorbitol	−	−	−	+
D-cellobiose, myo-inositol, N-acetyl-D-glucosamine, L-alanine, L-glutamic acid, D-gluconic acid, L-lactic acid, citric acid, D-saccharic acid	−	−	+	+

All strains were negative for erythrose, D-arabinose, L-xylose, β-methyl-D-xyloside, sorbose, dulcitol, α-methyl-D-glucoside, inulin, melezitose, starch, glycogen, xylitol, D-turanose, D-tagatose, D-fucose, L-fucose, L-arabitol, L-arginine, L-lysine, L-ornithine, sodium thiosulfate, urea, stachyose, D-salicin, N-acetyl-D-galactosamine, N-acetyl neuraminic acid, 8% w/v NaCl, D-mannitol, D-aspartic acid, D-serine, L-pyroglutamic acid, p-hydroxy-phenylacetic acid, D-lactic acid methyl ester, α-keto-glutaric acid, nalidixic acid, potassium tellurite, Tween 40, γ-amino-butyric acid, α-hydroxy-butyric acid, β-hydroxy-D, L-butyric acid, α-keto-butyric acid, acetoacetic acid, propionic acid, sodium butyrate, and sodium bromate. All strains were positive (at least weakly) for inositol, D-glucose, ribose, D-xylose, galactose, fructose, mannose, rhamnose, mannitol, N-acetylglucosamine, arbutin, maltose, trehalose, o-nitro-phenyl-β-D-galactopyranoside, L-tryptophane, Kohn’s gelatin, D-mannitol, sucrose, 1% w/v NaCl, α-D-glucose, D-mannose, D-fructose, D-galactose, D-fucose, L-rhamnose, 1% w/v sodium lactate, D-glucose-6-PO_4_, D-fructose-6-PO_4_, troleandomycin, rifamycin SV, Niaproof 4, D-galacturonic acid, L-galactono-1,4-lactone, D-glucuronic acid, glucuronamide, vancomycin, tetrazolium violet, and tetrazolium blue. All data are from the present study; +, positive; W, weakly positive; −, negative.

The fatty acid analysis revealed that all strains contained C_12:0_, C_14:0_, C_16:0_, C_17:0_ cyclo, summed feature 3 (C_16:1_ ω7c and/or C_16:1_ ω6c), and summed feature 8 fatty acids (C_18:1_ω6c and/or C_18:1_ω7c) as the major components (**Table 2**). The major cellular fatty acid profile (>5% of total) of strain MQR6^T^ was summed feature 3 (C_16:1_ω7c and/or C_16:1_ω6c), summed feature 8 (C_18:1_ω6c and/or C_18:1_ω7c), C_14:0_, C_16:0_, and C_17:0_ cyclo.

**Table 2:** The relative cellular fatty acid content (%) of strain MQR6^T^ and representative strains of closely related species of genus *Pantoea*.

Cellular fatty acid (%)	MQR6^T^	*P. vagans* DSM 23078^T^	*P. allii* DSM 25133^T^	*P. ananatis* ATCC 19321^T^
C_12:0_	4.4	6.4	12.0	11.0
C_14:0_	5.4	7.3	6.5	5.5
C_16:0_	24.0	25	19	18.0
C_17:0_ cyclo acid	12.0	7.2	2.1	1.9
Summed feature 3	23.0	32.0	31.0	22.0
Summed feature 8	17.0	9.3	8.3	10.0

All data are from this study. Fatty acid values are given as a percentage of the total peak area. Partial values lower than 1% are not shown in the table.

Summed features represent groups of two fatty acids that cannot be separated using the MIDI Sherlock system. Summed feature 3  =  C_16:1_ω6c and/or C_16:1_ω7c; Summed feature 8  =  C_18:1_ω6c and/or C_18:1_ω7c.

### 3.2 Phylogenetic analysis of 16S rRNA

**Figure 2:**
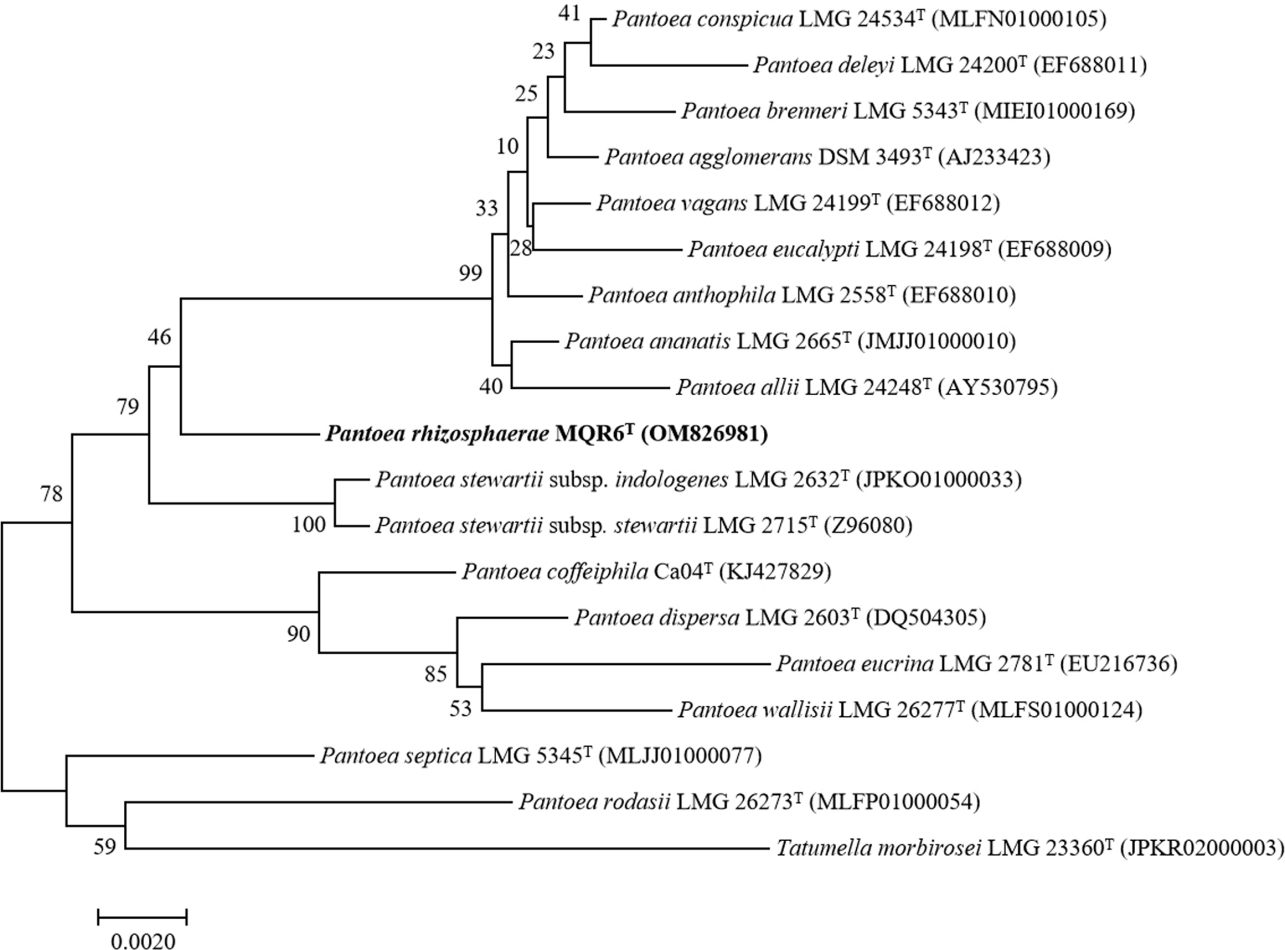
Neighbour-joining phylogenetic tree based on the 16S rRNA gene sequences of *Pantoea rhizosphaerae* strain MQR6^T^ and other closely related species. The significance of each branch is indicated by a bootstrap value (%) calculated for 1000 subsets. Genbank accession numbers are given in parentheses. Bar = 0.0020 nucleotide substitutions per position.

The 16S rRNA gene sequence (1440 bp) of strain MQR6^T^ was deposited in GenBank under the accession number OM826981. Based on the analysis of the EzBioCloud database, the strain MQR6^T^ was related closely to *P. vagans* DSM 23078^T^ (98.47%) and *P. ananatis* CICC 10283^T^ (98.51% similarity). Phylogenetic trees were reconstructed using the neighbor joining, maximum likelihood, and maximum parsimony methods (**Figure 2**, **Figures S1**, **S2**). All three treeing methods yielded similar phylogeny. Strain MQR6^T^ was located within the genus *Pantoea* and had a separate clade, indicating that strain MQR6^T^ represented a member of a novel species of genus *Pantoea*.

### 3.3 Whole-genome analysis

**Figure 3:**
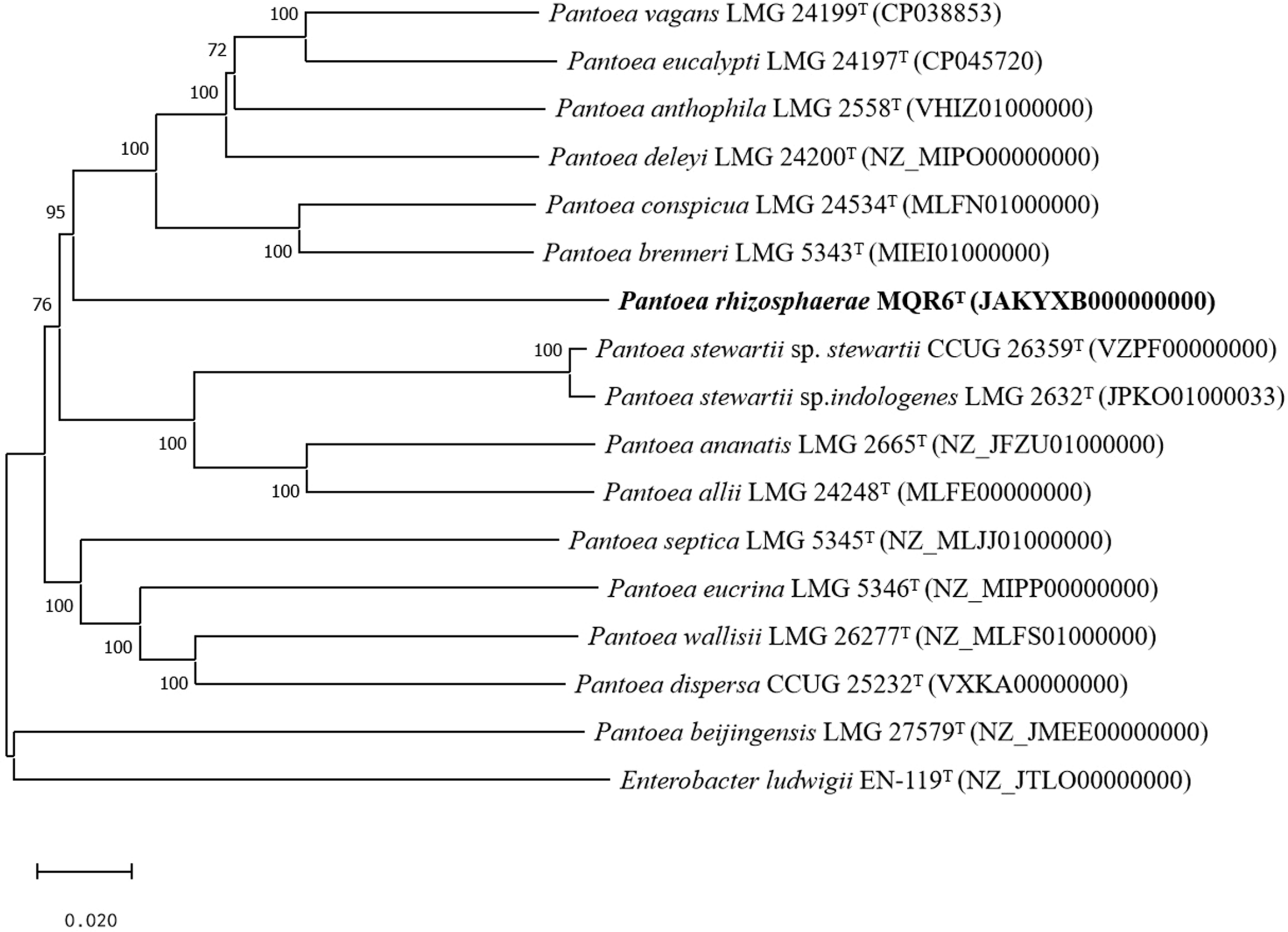
Tree inferred with FastME 2.1.6.1 from GBDP distances calculated from genome sequences of closely related species. The branch lengths are scaled in terms of GBDP distance formula d5. The numbers above branches are GBDP pseudo-bootstrap support values when >60% from 100 replications. GenBank genome accession numbers are given in parentheses. Bar = 0.0020 nucleotide substitutions per position.

**Figure 4:**
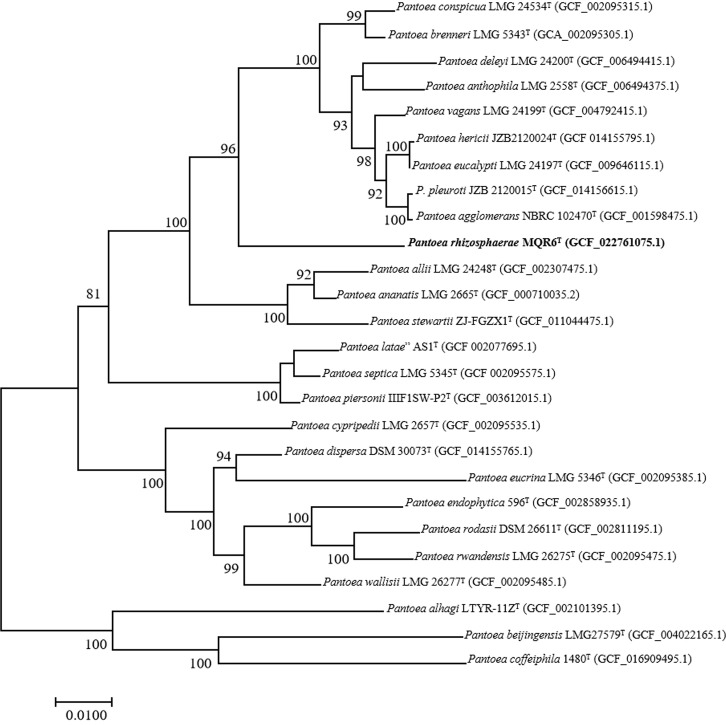
Phylogenomic tree inferred from the concatenation of 120 single-copy bacterial marker genes showing the phylogenetic position of strain MQR6^T^. A number on nodes represent bootstrap values based on the 1000 replications. Bootstrap values (≥ 70.0%) are shown at branch nodes. RefSeq assembly accession numbers are given in parentheses. Bar represents 0.01 nucleotide substitutions per position.

A total of 7,989,160 reads were obtained from genome sequencing of strain MQR6^T^, yielding a genome of 7,674,100 reads in length. The genome was predicted to contain a total of 4548 genes, which included 4473 protein-coding genes, 4 rRNA genes and 71 tRNA genes. There were 31 contigs in strain MQR6^T^. The genomic DNA G+C content of strain MQR6^T^ was 51.3%. The dDDH values between strain MQR6^T^ and the type strains of the genus *Pantoea* were 19.5-23.3%, and the average nucleotide identity based on blast (ANIb) between them was lower than 79.7% (**Table 3**). The phylogenomic tree based on the Type (Strain) Genome Server (TYGS) web also revealed the distinct phylogeny of strain MQR6^T^ and its close relationship with *P. ananatis* LMG 2665^T^, *P. allii* LMG 24248^T^ and *P. dispersa* DRS002603^T^ (**Figure 3**). In a phylogenetic tree based on 120 single-copy genes and the whole genome, strain MQR6^T^ forms a separate evolutionary branch (**Figure 4**). We conducted a preliminary analysis of the pan-genome, which showed that 1350 shared orthologous coding sequences were clustered into the core genome of *Pantoea*, 65,787 were represented in the accessory genome, and 8030 were identified as strain-unique genes. The total number of genes increased in the pan-genome of *Pantoea* with the rise in the analyzed genome number, suggesting that the pan-genome was open (**Figure S3**). The previous reports showed that the gene number in the core genomes was highly conserved, while many strain-unique genomes and accessory genomes were thought to contribute to species diversity, indicating that species in the genus *Pantoea* were multifarious.

**Table 3:** Average Nucleotide Identity based on blast (ANIb) and digital DNA-DNA Hybridization (dDDH) values of strain MQR6^T^ compared with all other tested *Pantoea* strains.

Strains	Genbank number	ANIb (%)	dDDH (%)
*P. conspicua*	GCF_002095315.1	79.6	23.2
*P. brenneri*	GCA_002095305.1	79.6	23.3
*P. agglomerans*	GCF_001598475.1	79.1	22.9
*P. pleuroti*	GCF_014156615.1	79.0	22.9
*P. vagans*	GCF_004792415.1	79.0	22.8
*P. hericii*	GCF_014155795.1	79.0	22.6
*P. eucalypti*	GCF_009646115.1	78.8	22.6
*P. anthophila*	GCF_006494375.1	78.6	22.5
*P. deleyi*	GCF_006494415.1	78.5	22.5
*P. septica*	GCF_002095575.1	77.6	21.2
*P. allii*	GCF_002307475.1	77.5	21.6
*P. ananatis*	GCF_000710035.2	77.5	21.4
*P. piersonii*	GCF_003612015.1	77.5	21.4
*P. latae*	GCF_002077695.1	77.4	21.2
*P. endophytica*	GCF_002858935.1	77.3	21.1
*P. stewartii*	GCF_011044475.1	77.2	21.4
*P. cypripedii*	GCF_002095535.1	77.2	21.1
*P. dispersa*	GCF_014155765.1	76.8	21.0
*P. rodasii*	GCF_002811195.1	76.8	20.9
*P. wallisii*	GCF_002095485.1	76.6	21.0
*P. rwandensis*	GCF_002095475.1	76.5	20.7
*P. eucrina*	GCF_002095385.1	75.7	20.4
*P. alhagi*	GCF_002101395.1	74.2	20.3
*P. beijingensis*	GCF_004022165.1	73.5	20.1
*P. coffeiphila*	GCF_016909495.1	73.4	19.5

The proposed and generally accepted species boundary for ANIb and dDDH values are 95~96 and 70%, respectively. The Genbank assembly accession number of strain MQR6^T^ is GCA_022761075.1 (latest).

The whole-genome shotgun sequencing output has been deposited at DDBJ/ENA/GenBank under the accession JAKYXB000000000. The version described in this paper is JAKYXB010000000.

### 3.4 Identification of genes responsible for plant growth-promoting characteristics of strain MQR6^T^

Functional analysis of the strain MQR6^T^ genome identified genes associated with the solubilization of phosphate and production of IAA, siderophores and phytohormones that are conducive to plant growth promotion. Eight key genes responsible for IAA production were found in MQR6^T^ genome, including seven genes related to tryptophan operon (*trpS*, *trpB*, *trpH*, *trpR*, *trpA*, *trpC*, *trpE*) and the *ipd C* gene encoding indole pyruvate decarboxylase (**Table S1**). These results indicate that IPyA pathway may be the main pathway for IAA production in the strain MQR6^T^.

The phosphonate-related *phn* gene cluster is responsible for the release of biologically available phosphate through the bacterial degradation of phosphonates. Our study revealed that strain MQR6^T^ carries several *phn* genes, including *phnN*, *phnM etc.*, showing the capacity to hydrolyze phosphonate into phosphate and alkane (**Table S2**).

Gluconic acid (GA) is an organic acid that is largely responsible for the solubilization of mineral phosphates. GA biosynthesis is carried out by glucose-1-dehydrogenase along with its co-factor pyrrolo-quinolone quinine. Accordingly, MQR6^T^ genome annotation indicated the presence of several genes related to gluconic acid biosynthesis and its co-factor genes, including *pqqBDEF* and *gcd*. Another organic acid identified in the strain MQR6^T^ that is relevant to the phosphate-solubilizing trait is 2-ketogluconic acid produced by gluconate 2-dehydrogenase alpha/beta chain and 2-keto-D-gluconate reductase. Moreover, the strain MQR6^T^ was found to produce other organic acids such as lactic, acetic, glycolic, and succinic (**Table S3**).

Genomic study showed that MQR6^T^ may synthesize an enterobactin siderophore involving the *entABCEF* genes. The siderophore is then exported from the cell using *entS* and is responsible for recovering iron by complexing. Having several siderophore receptor genes (**Table S4**), strain MQR6^T^ may take up siderophores produced by other organisms as well.

### 3.5 Quantification of P solubilization by strain MQR6^T^

**Figure 5:**
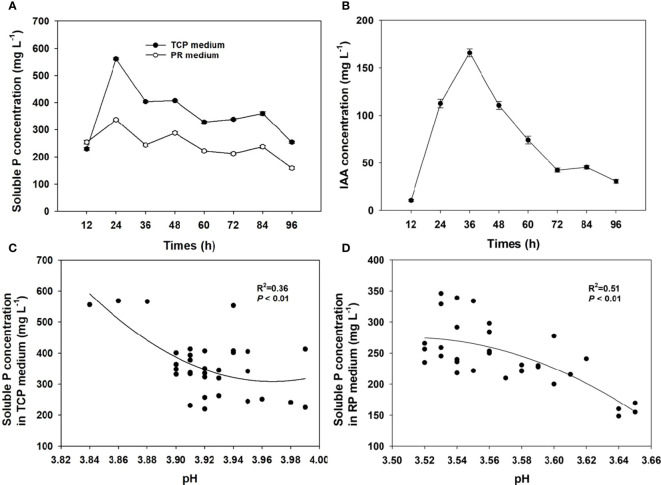
The solubilization of P in the TCP and RP media **(A)** and production of IAA **(B)** in the TSB medium with strain MQR6^T^ at 12-h intervals, and the relationship between soluble P concentration and pH in the TCP and RP media **(C, D)**. Means ± SE (where larger than the symbol), n=4. TCP, tricalcium phosphate; RP, rock phosphate.

Clear halozones were formed around the colonies of strain MQR6^T^ on inorganic phosphate media, with solubilization index (SI) values from 3.20 to 3.98 (**Figure 1C**). The strain MQR6^T^ was able to solubilize water-insoluble TCP and powdered rock phosphate (RP); however, amount of P solubilized was significantly higher in the TCP medium as compared to the RP medium (**Figure 5A**). The soluble P concentration in the TCP medium ranged between 232 and 559 mg L^-1^, with variations over time. By contrast, the soluble P concentration in the RP medium exhibited a range of 159–339 mg L^-1^. The highest concentration of soluble P in the two media was observed after 24 h incubation, then P solubilization gradually decreased over time.

The pH of the culture filtrates of strain MQR6^T^ decreased from an initial level of 7.22 to 3.84 in the TCP medium and to 3.52 in the RP medium (**Figures 5C, D**). There was a negative correlation between soluble P concentration and pH value of the TCP medium (R^2^ = 0.36, *P* < 0.01) and RP medium (R^2^ = 0.51, *P* < 0.01).

### 3.6 Quantification of IAA production and screening of siderophore

Strain MQR6^T^ showed pink color reaction with Salkowski reagent which indicated the production of IAA (**Figure 1F**). The highest (166 mg L^-1^) concentration of IAA in the medium with L-tryptophan was observed after 24 h incubation (**Figure 5B**). The siderophore production was detected using CAS, showing orange colonies after incubation due to siderophore-dependent removal of Fe from the dye (**Figure 1E**), indicating the capacity of strain MQR6^T^ to exude siderophore.

### 3.7 Plant growth of and nutrient optake by *A. truncatum* seedlings

**Figure 6:**
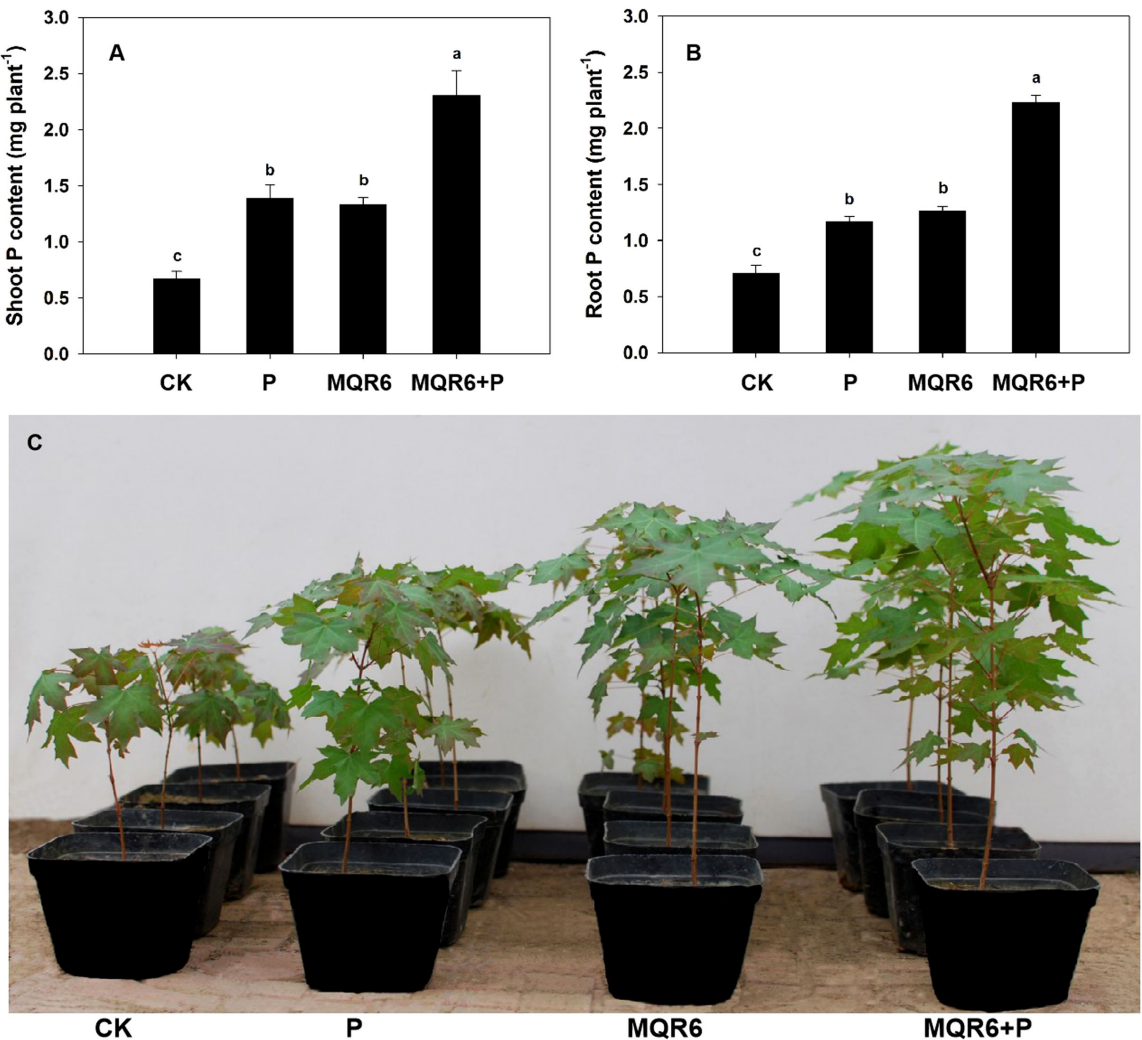
Effect of strain MQR6^T^ and supply of P (as KH_2_PO_4_) on P accumulation in shoots **(A)** and roots **(B)** and shoot growth **(C)** of *Acer truncatum* 290 days after sowing. Means + SE, n=4. CK, non-inoculated and without the application of P fertilizer; P, addition of P only; MQR6, inoculation with strain MQR6^T^ only; MQR6+P, inoculation with MQR6 plus the application of P. Different lowercase letters denote significant difference (*P* ≤ 0.05) among treatments.

Inoculation with strain MQR6^T^ significantly enhanced the shoot and root growth, dry weight accumulation and P uptake of *A. truncatum* seedlings compared to those grown in non-inoculated soils (**Table 4** and **Figure 6**). Compared with the non-inoculated control treatment, plant parameters in the MQR6^T^ inoculation treatment increased by 47% (height), 53% (trunk diameter), 15% (SPAD chlorophyll content), 100% (shoot dry weight) and 133% (root dry weight). Phosphorus accumulations in shoots and roots were, respectively, 102% and 79% greater in the MQR6^T^ inoculated treatment than those in the non-inoculated control. Similarly, P addition alone significantly increased plant height, trunk diameter, dry weight and P uptake when compared to the non-inoculated control treatment. Moreover, combined P addition and inoculation treatment significantly increased plant height, trunk diameter, biomass accumulation and P uptake when compared to the control, the P addition only and the inoculation only treatments. No significant difference was found in root/shoot ratio among the four treatments, and there was no significant difference in shoot and root biomass, SPAD chlorophyll content and P uptake between treatments with P addition and inoculation only.

**Table 4:** The effect of inoculation with strain MQR6^T^ and supply of P on the growth of *Acer truncatum* seedlings.

Parameters	CK	P	MQR6	MQR6+P
Plant height (cm)	15 ± 1d	19 ± 1c	22 ± 1b	27 ± 1a
Trunk diameter (mm)	1.5 ± 0.1d	2.0 ± 0.04c	2.3 ± 0.1b	2.6 ± 0.1a
Shoot dry weight (g plant^-1^)	0.4 ± 0.04c	0.7 ± 0.1b	0.8 ± 0.04b	1.3 ± 0.1a
Root dry weight (g plant^-1^)	0.3 ± 0.03c	0.6 ± 0.03b	0.7 ± 0.02b	1.1 ± 0.1a
Root/shoot ratio	0.9 ± 0.1a	0.9 ± 0.1a	0.9 ± 0.03a	0.9 ± 0.1a
SPAD chlorophyll content	26 ± 1c	28 ± 0.4b	30 ± 0.3b	31 ± 1a

Each value is the mean of four replicates ± SE. Different letters in each row denote significant difference among treatments. CK, non-inoculated and without the application of P fertilizer; P, addition of P only; MQR6, inoculation with strain MQR6^T^ only; MQR6+P, inoculation with MQR6 plus the application of P.

## 4 Discussion

Microorganisms capable of producing clear zones (halos) around the colonies growing on solid medium were selected as potential phosphate solubilizers (Gupta et al., 1994) and were screened repeatedly by a plate assay method using either Pikovskaya agar medium or TCP medium (Yu et al., 2011). Generally, the preliminary capacity of PSM to solubilize the insoluble phosphate was determined by the SI value (Li et al., 2020). Previous study showed that among 78 isolated strains, the majority exhibited a low index (SI<2.0), but eight strains had intermediate values (2.0<SI<4.0) and none of the strains showed a high solubility index (SI>4.0) for CaHPO_4_ solubilization (Marra et al., 2012). Similar results were also reported with yeast strains exhibiting a P-solubilization potential with SI ranging from 1.2 to 2.8; and a significant positive correlation was found between the solubilized amounts of P and the P solubilization index (Hesham and Mohamed, 2011). By contrast, other study observed isolated *Pseudomonas* sp. strain PSB-2 exhibited good solubilization of TCP with a high SI (>4.0) (He and Wan, 2022). In the present study, MQR6^T^ was isolated from the rhizosphere soil of *A. truncatum*, and showed clear halos of solubilizing TCP with the largest SI value of 3.98. Hence, the strain was found to be efficient phosphate solubilizer, and was selected for further evaluation.

In the present study, the general characteristics of MQR6^T^ corresponded with the genus *Pantoea* as described by Brady et al. (Brady et al., 2010): Gram-negative, rod-shaped, facultatively anaerobic, non-spore-forming, and commonly motile by means of peritrichous flagella (**Figure 1**). Analysis of 16S rRNA and complete genome sequencing and the phylogenetic trees reconstructed by using different methods found that the isolate belonged to *Pantoea* spp. and had a separate clade (**Figures 2**–**4**). In addition, the main fatty acids were hexadecanoic (C_16:0_), cyclo-heptadecanoic (C_17:0_ cycle) and summed feature 3 acids (containing C_16:1_ω6c and/or C_16:1_ω7c) (**Table 2**), in accordance with characteristics of *Pantoea* spp. (Mergaert et al., 1993). The genomic DNA G+C content of strain MQR6^T^ was 51.3 mol%, which is consistent with the DNA G+C contents ranging from 49.7 to 61.3 mol% of other members of the genus *Pantoea* (Brady et al., 2010). The dDDH values between strain MQR6^T^ and the type strains of the genus *Pantoea* were 19.5-23.3%, well below the dDDH standard cut-off value of 70% (Wang et al., 2020). On the basis of phylogenetic, physiological and chemotaxonomic characteristics, strain MQR6^T^ represents a novel species within the genus *Pantoea*, for which the name *Pantoea rhizosphaerae* sp. nov. is proposed.

The bacterial genus *Pantoea* comprises many versatile species that have been isolated from aquatic and terrestrial environments, living in association with plants, insects, humans, and animals (Castagno et al., 2011; Dutkiewicz et al., 2016; Luziatelli et al., 2020). Some isolates possess the capacities for nitrogen fixation, P solubilization, antibiotic production, and plant growth-promotion, and are being explored currently for agricultural applications (Chen and Liu, 2019; Luziatelli et al., 2020). Strain of P-dissolving *Pantoea agglomerans* R-42 was isolated from soybean rhizosphere had a marked insoluble phosphate-solubilizing activity (Son et al., 2006). In a different study, 50 PSB strains were isolated from the rhizosphere of *Lotus tenuis* grown in low-P soils (< 3 mg kg^-1^ of the available P) of the Salado River Basin; based on 16S rRNA gene sequencing, they belonged to *Pantoea*, *Erwinia, Pseudomonas, Rhizobium*, and *Enterobacter* genera; the most efficient isolate was identified as *Pantoea eucalypti*, a novel species of plant growth-promoting rhizobacteria (Castagno et al., 2011). Phosphate-solubilizing bacteria are known to be able to solubilize different forms of inorganic phosphates. The isolates *Pantoea agglomerans* ZB and *Pantoea* sp. S32 solubilized TCP, CaHPO_4_, RP, AlPO_4_, and FePO_4_ (Castagno et al., 2011; Chen and Liu, 2019; Li et al., 2020). In the present study, the tested strain had the capacity to solubilize inorganic TCP and RP, and the solubilization of TCP was about 60% more effective than RP. These results indicated that the tested strain MQR6^T^ may be effective to release soluble P from insoluble TCP in calcareous soil, which can be a source of P for plant growth.

Some studies have showed that P solubilization by bacterial strain was significantly influenced by the sources of P used in the media (Son et al., 2006; Panhwar et al., 2011). In the work presented here, we found that the concentration of soluble P was lower in the RP medium than the TCP medium, even though the pH was slightly lower in the RP than TCP medium. Similarly in the other study, a larger drop in pH was noted in the RP that TCP medium (Panhwar et al., 2011). The lower concentration of solubilized P in RP medium (**Figure 5D**) may be due to hampering (or even cessation) of bacterial P-solubilization activity at low pH. The influence of initial pH on the growth of MQR6^T^ was investigated in the pH range of 3.0 to 12.0, and pH levels below 4 resulted in a large reduction of bacterial population (data not shown). A clear relationship was established between bacterial growth and P solubilization in broth cultures (Panhwar et al., 2011). In addition, the rock phosphate has low P solubility as compared to calcium phosphate. This indicates decreased bacterial population and low pH may be associated with lower P solubilization in RP than TCP media.

Low-molecular-weight organic acids, such as acetic, oxalic and gluconic, have a high potential to solubilize water-insoluble inorganic phosphates (Hassan et al., 2019; Chen et al., 2020). Gluconic and 2-keto gluconic acids produced by bacteria play an important role in weathering and solubilization of phosphate in soil, acting as Ca^2+^ chelators and providing the acidification of the external environment to dissolve the sparingly soluble calcium phosphates (Yu et al., 2011). Furthermore, the glucose dehydrogenase (*gcd*) gene, coding for the first enzyme in the direct oxidation pathway, contributes significantly to mineral phosphate solubilization by the plant growth-promoting rhizabacteria (Shariati et al., 2017; He and Wan, 2022). Similarly, our study revealed that the genes related to gluconic and 2-keto-D-gluconic acid production were found in the strain MQR6^T^, including *gcd*, *gdh*, *pqq*, and *bet* (**Table S3**). Several genes responsible for biosynthesis of organic acids glycolic, acetic and succinic were also found in the genome of the strain MQR6^T^. Moreover, many PSM lower the pH of the medium by H^+^ extrusion (Surapat et al., 2013). A significant linear correlation was observed between culture pH and P solubilized from inorganic phosphate (Yu et al., 2011). Similar results were found between pH and solubilized P concentration in both the media tested in the present study (**Figures 5C, D**). Inoculation with strain MQR6^T^ may induce excretion of organic acid anions and H^+^ (via separate mechanisms), increasing the concentration of organic ligands and lowering the rhizosphere pH, enhancing a capacity to mobilize P in the rhizosphere.

Inoculation with MQR6^T^ showed a positive effect on plant height, trunk diameter, dry biomass, and P accumulation of *A. truncatum* Bunge seedlings in comparison with non-inoculated control (**Table 4**, **Figure 6**). Similar growth-promoting effects such as enhanced plant growth, dry weight accumulation and P uptake were also exhibited in sugar maple (*A. saccharum* Marsh.) seedlings inoculated with the strain MQR6^T^ (data not shown). The possible explanations for increased growth and P accumulation under inoculation with MQR6^T^ may include: (і) direct contribution to the mobilization of soil P by influencing rhizosphere pH and exudation of organic acid anions and phosphatases (Hinsinger, 2001; Shariati et al., 2017); (ii) the enhancement of root growth, including increased root length and root surface area, may be associated with improved spatial nutrient acquisition and chemical mobilization of P nutrients (Ma et al., 2021); (iii) several genes related to tryptophan operon (*trpS*, *trpB*, *trpH*, *trpR*, *trpA*, *trpC*, *trpE*) and one *ipd C* gene encoding indole pyruvate decarboxylase found in the strain MQR6^T^ genome may affect the amount of IAA produced, influencing root growth and water and nutrient acquisition (Radwanski and Last, 1995); (iv) more than 10 phosphonate-related *Phn* genes found in the strain MQR6^T^ genome may enable sufficient phosphate uptake to support bacterial growth (Stasi et al., 2019); and (v) several genes for siderophore receptors, such as *entA*, *entB* and *entC*, found in the strain MQR6^T^ genome are responsible for iron recovery by complex formation (Saha et al., 2013). Furthermore, the transcriptomic responses of *A. truncatum* roots inoculated with MQR6^T^ are currently under investigation to decipher the relevant plant-microbe interactions.

The application of P in combination with MQR6^T^ significantly improved the plant growth, dry weight and P uptake of *A. truncatum* seedlings compared with the P alone and MQR6^T^ alone treatments (**Figure 6**). Similarly, the most pronounced beneficial effect on growth of walnut plants was observed in the treatment with both inoculation and P addition, which was attributed partly to an increase in the population of PSB in the rhizosphere (Yu et al., 2011). Addition of inorganic P to the inoculated soil further stimulated growth of bacteria and raised the total population of PSB (Yu et al., 2011). Other study reported that competition for P did exist among PSB, arbuscular mycorrhizal (AM) fungi and plant, especially in a low-P soil, and the competition would have been alleviated by supplying an optimal amount of P to the soil (Zhang et al., 2014). In the present study, addition of 50 mg P kg^-1^ soil may be beneficial not only for proliferation and survival of strain MQR6^T^ but also for growth of *A. truncatum* seedings. In addition, the interactions between MQR6^T^ and P fertilizer could cause a synergistic effect that allowed improved root and shoot growth of *A. truncatum*. However, the molecular and physiological mechanism behind the effect of inoculation with MQR6^T^ with or without P application on *A. truncatum* growth, especially in the field conditions, needs to be investigated further.

## 5 Conclusion

In conclusion, the new strain MQR6^T^, isolated from *A. truncatum* rhizosphere, was demonstrated to belong to a new *Pantoea rhizosphaerae* species on the basis of phylogenetic, physiological and chemotaxonomic characteristics. The *P*. *rhizosphaerae* strain MQR6^T^ exhibited a high capacity to solubilize phosphate and produce IAA and siderophores, associated with the relevant genes found in the genome. The significant enhancement in shoot and root growth and P uptake of *A. truncatum* seedlings inoculated with MQR6^T^ confirmed that it is a growth-promoting rhizobacterium, potentially providing a basis for a new inoculant biofertilizer for *A. truncatum* cultivation, especially in low-P soils.

## Data availability statement

The datasets presented in this study can be found in online repositories. The names of the repository/repositories and accession number(s) can be found below: https://www.ncbi.nlm.nih.gov/genbank/, JAKYXB000000000.

## Author contributions

QM, XX and XZ designed and performed experiments, analyzed and interpreted data, and wrote the manuscript. SH performed genomic analysis, XingW assisted with experimental design and performed the experiments, LC assisted with experimental design and supervised the study. XinghW and SP assisted with performing the experiments and analyzing the data. ZR revised the manuscript critically. XX and XZ supervised the study, designed experiments and reviewed the manuscript. All authors contributed to the article and approved the submitted version.
