# Supplementary material for: Isolation and characterization of phosphate-solubilizing bacterium Pantoea rhizosphaerae sp. nov. from Acer truncatum rhizosphere soil and its effect on Acer truncatum growth
Source: Front Plant Sci. 2023 Jul 14;14:1218445. doi: 10.3389/fpls.2023.1218445 (PMC10375718; doi:10.3389/fpls.2023.1218445)
Supplement: Supplementary file 1 [file DataSheet_1.doc]

**Figure captions**

**FIGURE S1** Maximum-likelihood phylogenetic tree based on the 16S rRNA gene sequences of strain MQR6^T^ and other closely related species. The significance of each branch is indicated by a bootstrap value (%) calculated for 1000 subsets. Genbank accession numbers are given in parentheses. Bar denotes 0.0020 nucleotide substitutions per position.

**FIGURE S2** Maximum-parsimony phylogenetic tree based on the 16S rRNA gene sequences of strain MQR6^T^ and other closely related species. The significance of each branch is indicated by a bootstrap value (%) calculated for 1000 subsets. Genbank accession numbers are given in parentheses. Bar = 5 nucleotide substitutions per position.

**FIGURE S3** The relationship between the number of genomes and the number of gene families in the pan genome and the core genome of *Pantoea*.

*Pantoea conspicua* LMG 24534^T^ (MLFN01000105)

*Pantoea deleyi* LMG 24200^T^ (EF688011)

*Pantoea brenneri* LMG 5343^T^ (MIEI01000169)

*Pantoea agglomerans* DSM 3493^T^ (AJ233423)

*Pantoea vagans* LMG 24199^T^ (EF688012)

*Pantoea eucalypti* LMG 24198^T^ (EF688009)

*Pantoea anthophila* LMG 2558^T^ (EF688010)

*Pantoea ananatis* LMG 2665^T^ (JMJJ01000010)

*Pantoea allii* LMG 24248^T^ (AY530795)

***Pantoea rhizosphaerae* MQR6^T^ (OM826981)**

*Pantoea stewartii* subsp. *indologenes* LMG 2632^T^ (JPKO01000033)

*Pantoea stewartii* subsp. *stewartii* LMG 2715^T^ (Z96080)

*Pantoea coffeiphila* Ca04^T^ (KJ427829)

*Pantoea dispersa* LMG 2603^T^ (DQ504305)

*Pantoea eucrina* LMG 2781^T^ (EU216736)

*Pantoea wallisii* LMG 26277^T^ (MLFS01000124)

*Pantoea septica* LMG 5345^T^ (MLJJ01000077)

*Pantoea rodasii* LMG 26273^T^ (MLFP01000054)

*Tatumella morbirosei* LMG 23360^T^ (JPKR02000003)

70

86

92

61

85

99

42

46

42

42

99

27

25

16

0.0020

**Figure S1**

*Pantoea conspicua* LMG 24534^T^ (MLFN01000105)

*Pantoea deleyi* LMG 24200^T^ (EF688011)

*Pantoea brenneri* LMG 5343^T^ (MIEI01000169)

*Pantoea agglomerans* DSM 3493^T^ (AJ233423)

*Pantoea vagans* LMG 24199^T^ (EF688012)

*Pantoea eucalypti* LMG 24198^T^ (EF688009)

*Pantoea anthophila* LMG 2558^T^ (EF688010)

*Pantoea ananatis* LMG 2665^T^ (JMJJ01000010)

*Pantoea allii* LMG 24248^T^ (AY530795)

***Pantoea rhizosphaerae* MQR6^T^ (OM826981**)

*Pantoea stewartii* subsp. *indologenes* LMG 2632^T^ (JPKO01000033)

*Pantoea stewartii* subsp. *stewartii* LMG 2715^T^ (Z96080)

*Pantoea coffeiphila* Ca04^T^ (KJ427829)

*Pantoea dispersa* LMG 2603^T^ (DQ504305)

*Pantoea eucrina* LMG 2781^T^ (EU216736)

*Pantoea wallisii* LMG 26277^T^ (MLFS01000124)

*Pantoea septica* LMG 5345^T^ (MLJJ01000077)

*Pantoea rodasii* LMG 26273^T^ (MLFP01000054)

*Tatumella morbirosei* LMG 23360^T^ (JPKR02000003)

49

30

12

15

68

39

25

37

98

46

100

35

89

95

84

89

5

**Figure S2**


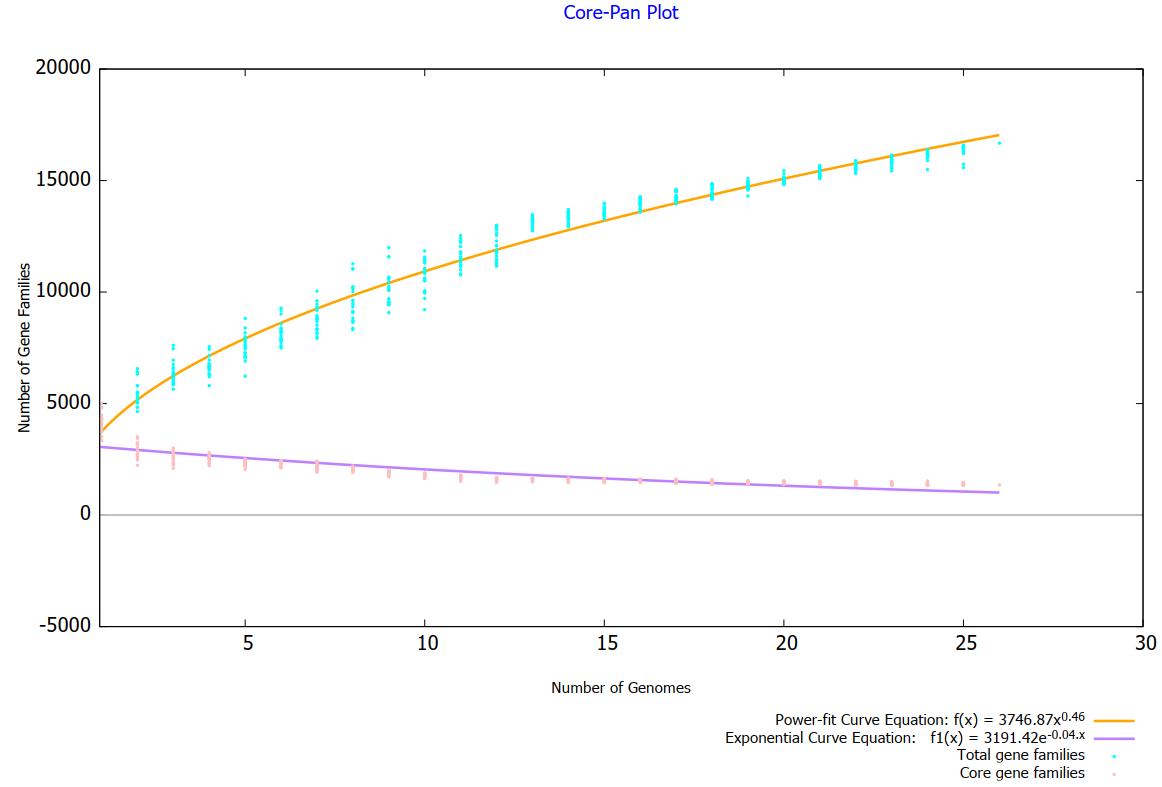


**Figure S3**
