# Supplementary material for: Isolation and characterization of phosphate-solubilizing bacterium Pantoea rhizosphaerae sp. nov. from Acer truncatum rhizosphere soil and its effect on Acer truncatum growth
Source: Front Plant Sci. 2023 Jul 14;14:1218445. doi: 10.3389/fpls.2023.1218445 (PMC10375718; doi:10.3389/fpls.2023.1218445)
Supplement: Supplementary file 2 [file Table_1.doc]

**TABLE S1 Genes related to IAA production in the strain MQR6^T^ genome.**

| **Protein ID** | **Gene annotation** | **KO/Gene ID** | **KEGG Gene number** | **EC Number** |
| --- | --- | --- | --- | --- |
| WP_215845394.1 | Tryptophanyl-tRNA synthetase | *trpS* | K01867 | 6.1.1.2 |
| WP_215846690.1 | The beta subunit is responsible for the synthesis of L- tryptophan from indole and L-serine | *trpB* | K01696 | 4.2.1.20 |
| WP_215846695.1 | PHP domain protein | *trpH* | K07053 | 3.1.3.97 |
| WP_215847683.1 | TrpR family transcriptional regulator, trp operon repressor | *trpR* | K03720 | - |
| WP_243077825.1 | The alpha subunit is responsible for the aldol cleavage of indoleglycerol phosphate to indole and glyceraldehyde-3-phosphate | *trpA* | K01695 | 4.2.1.20 |
| WP_243077826.1 | Belongs to the TrpC family | *trpC* | K13498 | 4.1.1.48, 5.3.1.24 |
| WP_243077827.1 | With component II, the glutamine amidotransferase catalyzes the formation of anthranilate from chorismate and glutamine | *trpE* | K01657, K13503 | 4.1.3.27 |
| WP_243077462.1 | Belongs to the TPP enzyme family | *ipdC* | K01568, K04103 | 4.1.1.1, 4.1.1.74 |

**TABLE S2 Genes related to phosphate & phosphonate transport capacity in the genome of strain MQR6^T^.**

| **Protein ID** | **Gene annotation** | **KO/Gene ID** | **KEGG Gene number** | **EC Number** |
| --- | --- | --- | --- | --- |
| WP_215845773.1 | Alkylphosphonate utilization operon protein PhnA | *phnA* | K06193 | - |
| WP_215846483.1 | phosphonate ABC transporter, periplasmic phosphonate binding protein | *phnD* | K02044 | - |
| WP_243077648.1 | Phosphonate C-P lyase system protein PhnG | *phnG* | K06166 | 2.7.8.37 |
| WP_243077649.1 | phosphonate C-P lyase system protein PhnH | *phnH* | K06165 | 2.7.8.37 |
| WP_243077650.1 | phosphonate metabolism | *phnI* | K06164 | 2.7.8.37 |
| WP_243077651.1 | Catalyzes the breakage of the C-P bond in alpha-D-ribose-1-methylphosphonate-5-phosphate (PRPn) forming alpha-D-ribose | *phnJ* | K06163 | 4.7.1.1 |
| WP_243077652.1 | phosphonate C-P lyase system protein PhnK | *phnK* | K05781 | - |
| WP_243077653.1 | phosphonate C-P lyase system protein PhnL | *phnL* | K05780 | 2.7.8.37 |
| WP_243077654.1 | phosphonate metabolism protein PhnM | *phnM* | K06162 | 3.6.1.63 |
| WP_243077655.1 | Catalyzes phosphorylation of ribose-1,5-bisphosphate to 5-phospho-D-ribosyl-α-1-diphosphate (PRPP) | *phnN* | K05774 | 2.7.4.23 |
| WP_243077656.1 | Metallo-beta-lactamase superfamily | *phnP* | K06167 | 3.1.4.55 |
| WP_243077657.1 | Part of the ABC transporter complex PhnCDE involved in phosphonates import. Responsible for energy coupling to the transport system | *phnC* | K02041 | 3.6.3.28 |
| WP_243077658.1 | phosphonate ABC transporter, inner membrane subunit | *phnE_2* | K02042 | - |
| WP_243077659.1 | phosphonate ABC transporter, inner membrane subunit | *phnE* | K02042 | - |
| WP_243078358.1 | Acetyltransferase (GNAT) domain | *phnO* | K09994 | - |
| WP_243078484.1 | Transcriptional regulator | *phnF* | K02043,K03482 | - |
| WP_243078766.1 | Glyoxalase/Bleomycin resistance protein/Dioxygenase superfamily | *phnB* | K04750 | - |
| WP_243078798.1 | UTRA | *phnR* | K03710 | - |
| WP_215848092.1 | Part of the ABC transporter complex PstSACB involved in phosphate import. Responsible for energy coupling to the transport system | *pstB* | K02036 | 3.6.3.27 |
| WP_215848095.1 | Part of the ABC transporter complex PstSACB involved in phosphate import | *pstS* | K02040 | - |
| WP_243077417.1 | Binding-protein-dependent transport systems inner membrane component | *pstC* | K02037 | - |
| WP_243077418.1 | Phosphate transport system | *pstA* | K02038 | - |
| WP_243077419.1 | Part of the ABC transporter complex PstSACB involved in phosphate import. Responsible for energy coupling to the transport system | *pstB* | K02036 | 3.6.3.27 |
| WP_243078422.1 | Phosphate | *pstS* | K02040 | - |
| WP_243079810.1 | Binding-protein-dependent transport system inner membrane component | *pstA* | K02038 | - |
| WP_243079811.1 | Probably responsible for the translocation of the substrate across the membrane | *pstC* | K02037 | - |

**TABLE S3** Gene annotations of strain MQR6^T^ genome related to its phosphate solubilization capacity.

| **Trait** | **Protein ID** | **Description** | **KO/Gene ID** | **KEGG Gene number** | **EC number** |
| --- | --- | --- | --- | --- | --- |
| Gluconic acid | WP_215847856.1 | Quinoprotein glucose dehydrogenase | *gcd* | K00117, K05358 | 1.1.5.2,1.1.5.8 |
|  | WP_215848420.1 | Belongs to the Glu Leu Phe Val dehydrogenases family | *gdhA* | K00261, K00262 | 1.4.1.3,1.4.1.4 |
|  | WP_215846860.1 | Ring cyclization and eight-electron oxidation of 3a-(2-amino-2-carboxyethyl)-4,5-dioxo-4,5,6,7,8,9-hexahydroquinoline-7,9-dicarboxylic-acid to PQQ | *pqqC* | K06137 | 1.3.3.11 |
|  | WP_243077985.1 | Coenzyme PQQ biosynthesis protein PqqF | *pqqF* | - | - |
|  | WP_243077986.1 | Biosynthesis protein E | *pqqE* | K06139 | - |
|  | WP_243077987.1 | May be involved in the transport of PQQ or its precursor to the periplasm | *pqqB* | K06136 | - |
|  | WP_243078499.1 | With PqqC converts a biosynthetic intermediate to pyrroloquinoline quinone | *pqqD* | K06138 | - |
|  |  |  |  |  |  |
| 2-keto-D-gluconic acid | WP_215846720.1 | Involved in the biosynthesis of the osmoprotectant glycine betaine. Catalyzes the reversible oxidation of betaine aldehyde to the corresponding acid | *betB* | K00130 | 1.2.1.8 |
|  | WP_243077848.1 | Involved in the biosynthesis of the osmoprotectant glycine betaine. Catalyzes the oxidation of choline to betaine aldehyde and betaine aldehyde to glycine betaine at the same rate | *betA* | K00108 | 1.1.99.1 |
|  | WP_243077849.1 | Repressor involved in choline regulation of the bet genes | *betI* | K02167 | - |
|  | WP_243077850.1 | BCCT, betaine/carnitine/choline family transporter | *betT* | K02168 | - |
|  |  |  |  |  |  |
| Glycolic acid | WP_215847327.1 | in *Escherichia coli* this enzyme forms a trimer of dimers that is allosterically inhibited by NADH and competitively inhibited by α-ketoglutarate | *gltA* | K01647 | 2.3.3.1 |
|  | WP_243077833.1 | Catalyzes the isomerization of citrate to isocitrate via cis-aconitate | *acnA* | K01681 | 4.2.1.3 |
|  | WP_215847611.1 | Belongs to the aconitase IPM isomerase family | *acnB* | K01682 | 4.2.1.3, 4.2.1.99 |
|  | WP_243078808.1 | Isocitrate lyase | *aceA* | K01637 | 4.1.3.1 |
|  | WP_243079253.1 | D-isomer specific 2-hydroxyacid dehydrogenase | *hprA* | K00018 | 1.1.1.29 |
|  | WP_243078129.1 | Catalyzes the formation of glycolate and glycerate from glyoxylate and hydroxypyruvate, respectively | *ghrA* | K12972 | 1.1.1.79, 1.1.1.81 |
|  |  |  |  |  |  |
| Acetic acid | WP_243078584.1 | Component of the pyruvate dehydrogenase (PDH) complex that catalyzes the conversion of pyruvate to acetyl-CoA | *aceE* | K00163 | 1.2.4.1 |
|  | WP_243078583.1 | The pyruvate dehydrogenase complex catalyzes the conversion of pyruvate to acetyl-CoA | *aceF* | K00627 | 2.3.1.12 |
|  | WP_243078778.1 | Acetyl-CoA synthetase | *acsA* | K01895 | 6.2.1.1 |
|  | WP_243077816.1 | Belongs to the iron-containing alcohol dehydrogenase family | *adhE* | K04072 | 1.1.1.1,1.2.1.10 |
|  | WP_243077633.1 | Belongs to the aldehyde dehydrogenase family | *-* | K00128 | 1.2.1.3 |
|  | WP_243078124.1 | Belongs to the aldehyde dehydrogenase family | *aldB* | K00128,K00138 | 1.2.1.3 |
|  |  |  |  |  |  |
| Succinate | WP_215847327.1 | In *Escherichia coli* this enzyme forms a trimer of dimers that is allosterically inhibited by NADH and competitively inhibited by α-ketoglutarate | *gltA* | K01647 | 2.3.3.1 |
|  | WP_215845465.1 | Catalyzes the reversible oxidation of malate to oxaloacetate | *mdh* | K00024 | 1.1.1.37 |
|  | WP_215845283.1 | Malate quinone oxidoreductase | *mqo* | K00116 | 1.1.5.4 |
|  | WP_243079183.1 | Malate quinone oxidoreductase | *mqo* | K00116 | 1.1.5.4 |
|  | WP_243078007.1 | Involved in the TCA cycle. Catalyzes the stereospecific interconversion of fumarate to L-malate | *fumC* | K01679 | 4.2.1.2 |
|  | WP_243079187.1 | Catalyzes the reversible hydration of fumarate to (S)-malate | *fumB* | K01676 | 4.2.1.2 |
|  | WP_215847324.1 | Belongs to the FAD-dependent oxidoreductase 2 family. FRD SDH subfamily | *sdhA* | K00239 | 1.3.5.1,1.3.5.4 |
|  | WP_215847323.1 | SdhA and B form the catalytic subcomplex and can exhibit succinate dehydrogenase activity in the absence of SdhC and D that are the membrane components and form cytochrome b556 | *sdhB* | K00240 | 1.3.5.1,1.3.5.4 |
|  | WP_215847326.1 | Succinate dehydrogenase | *sdhC* | K00241 | - |
|  | WP_215847325.1 | Succinate dehydrogenase hydrophobic membrane anchor subunit | *sdhD* | K00242 | - |

**TABLE S4 Genes related to siderophore biogenesis in the strain MQR6^T^ genome.**

| **Protein ID** | **Description** | **KO/Gene ID** | **KEGG Gene number** | **EC Number** |
| --- | --- | --- | --- | --- |
| WP_243078726.1 | Phosphopantetheine attachment site | *entB* | K01252 | 3.3.2.1, 6.3.2.14 |
| WP_215847804.1 | AMP-binding enzyme C-terminal domain | *entE* | K02363, K12238 | 2.7.7.58, 6.3.2.14 |
| WP_243078720.1 | Condensation domain | *entF* | K02364 | 6.3.2.14 |
| WP_243078723.1 | Major facilitator superfamily | *entS* | K08225 | - |
| WP_243078727.1 | KR domain | *entA* | K00216 | 1.3.1.28 |
| WP_243078725.1 | Chorismate binding enzyme | *entC* | K01851, K02361 | 5.4.4.2 |
| WP_243078724.1 | Periplasmic binding protein | *fepB* | K02016 | - |
| WP_243078722.1 | Belongs to the binding-protein-dependent transport system permease family. FecCD subfamily | *fepD* | K02015 | - |
| WP_215847800.1 | ATPases associated with a variety of cellular activities | *fepC* | K02013 | 3.6.3.34 |
| WP_243078721.1 | Belongs to the binding-protein-dependent transport system permease family. FecCD subfamily | *fepG* | K02015 | - |
| WP_243078719.1 | Receptor | *fepA* | K16089, K19611 | - |
